# Nutrition Leadership Development: Capacity-Building Initiatives in Iran and the Middle-East Region Since 2009

**DOI:** 10.3389/fpubh.2015.00184

**Published:** 2015-07-27

**Authors:** Azadeh Davari, Arash Rashidi, Jacques Antonius Baartmans

**Affiliations:** ^1^School of Leadership and Education Sciences (SOLES), University of San Diego, San Diego, CA, USA; ^2^Department of Food and Nutrition Policy and Planning Research, National Nutrition and Food Technology Research Institute, Faculty of Nutrition Sciences and Food Technology, Shahid Beheshti University of Medical Sciences, Tehran, Iran; ^3^The Leadership Group, Bloemendaal, Netherlands

**Keywords:** capacity building, empowerment, leadership competencies, nutrition discipline, nutrition professionals, Iran, the Middle-East

## Abstract

Personal and organizational performance is determined by commitment and both technical *and* general competencies, including leadership skills. Academia, however, mainly targets technical aspects in its curricular programs. On the other hand, the inter-disciplinary and multi-sector nature of Nutrition necessitates high levels of collaboration between stakeholders. Leadership development is therefore required in Nutrition. This paper describes the endeavor made in Iran and the Middle-East region, aiming at building leadership capacity among nutrition professionals. The empowered human resource is expected to facilitate nutrition security at the national and regional levels. Since 2007, the development process of the initiative has begun through research, bench marking, and consultation. The “learning organizations,” “leadership from inside-out,” and “transformational leadership” frameworks have been employed as underpinning theories. Main topics have been self-awareness, effective communication, shared visioning, trust building, creativity, and motivating. Outbound team-building activities and coaching have also been included. The first workshop of the Iranian Food and Nutrition Leadership Program was held in 2009 in Tehran. The experience expanded to the region as the Middle-East Nutrition Leadership Program (MENLP). The Ph.D. Nutrition programs (at four leading Universities) and Iranian Nutrition Society have been taken as other opportunity windows to develop leadership competencies. Biannual Iranian nutrition congresses have been used as the main media for advocacy purposes. High-satisfaction rates obtained following each training activity. In short, the initiative on “nutrition leadership development” has received growing investment and positive feedback in Iran. Continuous improvement of the initiative, establishment of active alumni networks, building MENLP regional platform, and integrating a monitoring and evaluation system are required to increase the investment returns.

## Introduction

Leadership, as one of the most talked about issues in the contemporary literature, has been looked at from different perspectives and has accordingly different definitions (1). There seem, however, a number of key constant themes in the majority of terminologies. Leadership is very dependent on a shared vision *and* on influencing. We may therefore simply define it as a *shared influence process* (1). This influence process may be led by a person, as leader, *or* by a charismatic common purpose, i.e., invisible leadership (2). The latter seems as the proper one to lead a “discipline” in both theoretical and practical aspects.

Development of leadership competencies has recently been justified for nutrition practitioners working in public health (3, 4) and clinic (5) settings. Moreover, echoing the recent discussions in *Nature* (6, 7) and our own experience, we raised the need for earlier leadership development among nutrition scientists (8). With no doubt, the fact arises from inter-disciplinary and multi-sector nature of nutrition sciences (9). The need to enhance leadership skills was previously shown among food technology professionals in Iran (10). Very recently, a pilot study found that nutrition professionals and discipline also required bridging leadership gaps in the West Asian region (11).

To build leadership capacity among young and promising nutrition professionals, Europe has created the oldest platform since mid-1990 (3). The initiative has been replicated in other continents and regions, e.g., Africa (for both Anglophone *and* Francophone countries), South-East Asia, and South America. We decided to make similar initiative in Iran (since 2009) and in the Middle-East region (since 2012).

This article is therefore going to shortly review the endeavor in Iran and the region to empower nutrition professionals through leadership capacity building. It will briefly explain different planning and administrative steps in establishment process of nutrition leadership development in the country, and will extract a number of lessons learned in the last 6 years, and a road map for future endeavors.

## Development of Capacity-Building Initiatives

Development of leadership capacity-building programs in Iran and the Middle-East region has followed a sequence of interconnected activities, in order to maximize the effectiveness of the initiative and to fit it to the leadership requirements of nutrition community. Cultural diversity, existing within and between the discipline and nations, has also considered when developing and delivering training programs. This is an endless endeavor; every step can bring changes to the programs and results in fine tuning. Below, the major steps taken to develop different leadership initiatives and programs for nutrition professionals are briefly reviewed.

### Visiting best practices

In order to bench-mark other nutrition leadership programs, participatory and observational visits were made to South-East Asian Nutrition Leadership Program (SEA-NLP) *and* European Nutrition Leadership Platform (ENLP) in 2007 and 2008, respectively. Lessons from other leadership development activities around the globe, such as Asia Pacific Leadership Program (APLP, East West Center, USA), were also collected.

### Research

Research is a pre-requisite to tailor-make a leadership development program. The first Iranian Food and Nutrition Leadership Program (IFNLP) was developed through a research project at National Nutrition and Food Technology Research Institute (NNFTRI), Tehran. The project was also integrated a mixed-method study on leadership skills among Iranian food science and technology professionals. The study showed that effective team building, communication, creativity, decision-making, holistic thinking and practicing, and trust building were *most-in-need* skills among the target group (10, 12).

Very recently, a mini survey using a structured questionnaire was conducted to examine essential elements of leadership in nutrition sector, as a “learning organization” (13), in selected countries of the West Asian region. The study also aimed to identify significant gaps in leadership competencies among nutrition professionals in the region. Countries of Iran, Lebanon, Saudi Arabia, and Turkey were selected as the representative *hubs* for nutrition discipline. Besides their longer history in nutrition education, research, and action compared to other member states, these countries could represent three main languages (i.e., Persian, Arabic, and Turkish), which were spoken in the region. The preliminary data have shown big gaps in leadership practices in nutrition discipline as expressed by respondents from academe (11).

We are presently doing a national study to explore the leadership requirements of community nutrition professionals who work for the health sector in Iran. Different sources, such as role statements, key informants, and a group of community nutrition managers, will be employed to maximize data triangulation (14), and to obtain both qualitative and quantitative data regarding the *real* and the *felt* leadership needs of the target audience. Findings from this exercise can provide educational needs to be considered in future training courses.

There are also a number of on-going research and administrative projects to come up with the best structural arrangements for a Middle-East Nutrition Leadership Platform, as well as the Alumni network and to find out educational needs in different nutrition branches (in a cell-to-society spectrum) and member countries in the region.

### Technical consultancies

The present initiatives of IFNLP, Middle-East Nutrition Leadership Program (MENLP), and other leadership programs have been based on extensive local and external consultancies. Efforts have been made to review and integrate views from both nutrition seniors/scholars *and* available school of thoughts in leadership development.

### Partnership

Along with other developmental activities, the leadership development initiative in Iranian nutrition community has built strong coalitions with a wide range of stakeholders as collaborating partners; leading nutrition schools and departments, The Iranian Ministry of Health, the UN agencies representatives in Iran (e.g., WHO and UNICEF), professional NGOs, and a number of big food industries have been stepped on the stage.

Iranian Food and Nutrition Leadership Program was developed at NNFTRI, Tehran. This institute, with its adjunct school, has been the first and leading nutrition education and research organization in the country. The establishment of NNFTRI was part of a regional endeavor made during 1960s to institutionalize nutrition discipline. Since 2009, the NNFTRI has supported and hosted many workshops of nutrition leadership initiatives/programs.

There was an initiative (2009–2011), *one-vision one-voice*, which was organized by the NNFTRI in partnership with food industry. The aim was to sensitize present nutrition leaders coming from academic and policy making departments about the importance and function of leadership in nutrition discipline, and to motivate and align participants for collaboration to develop a better future.

Another leadership development activity targeting young nutrition students and graduates was established by Iranian Nutrition Society (Professional NGOs). More than 90 members of the society participated in the nutrition leadership workshops, designed in four different levels, in the time period 2011–2013 in Tehran.

We also decided to integrate nutrition leadership into the established on-going programs. For example, an endeavor had already been made by leading nutrition departments to include leadership theme into the syllabus of the course *Managing Nutrition Programs*, Ph.D. Nutrition program. We found the opportunity to be invited by nutrition schools and departments at Shiraz, Tehran, Iran, and Shahid Beheshti Medical Universities to facilitate training sessions as part of the above course in the last 4 years.

In addition, several leadership trainings were organized for different target audiences, including hospital dieticians and community nutritionists in health care system. Workshop funds had totally or partially been provided by some major food and pharmaceutical companies.

However, making scientific and administrative coalition at “regional” level has been less developed, but seems promising. The NNFTRI hosted the first and the second training workshops of The MENLP in Tehran, together with Department of Nutrition and the representatives of WHO (May 8–10, 2012) and UNICEF (January 3–5, 2015), respectively.

All these partnerships have had invaluable conceptual, programmatic and financial inputs for the leadership initiative, providing the opportunity to experience multi-stakeholder processes and networking.

### Underpinning theories and constructs

The *learning organizations* theory discussed by Senge (13) has been used to build the main conceptual framework for leadership development initiative in nutrition. Based on that theory, *learning organizations* are organizations in which people strive to create the results that they desire by increasing capacity, nurturing new ways of thinking, and working together toward a shared vision. Senge identifies five essential elements within a learning organization: *system thinking*, *personal mastery*, *mental models*, *shared vision*, and *team learning*. *System thinking* involves a way of thinking and explanation language for understanding forces and interactions that control a system’s behavior. *Personal mastery* involves creation of an enabling environment and expansion of personal capacities to create desirable outcomes toward the goals. *Mental models* involve continuous reflection upon, analysis, clarification, and improvement of internal pictures of the world by each employee within organization to improve personal actions and decisions. *Shared vision* involves commitment and shared image within a workgroup of desired future as well as general agreement on principles and guiding practices to support journey to such future. *Team learning* involves relevant thinking skill, which enables a working group to develop collective intelligence and ability greater than sum of members’ talents and performance.

Within each element, there are leadership skills or competencies demonstrated by the individuals in the organization. For example, behaviors, such as being open to criticism, admitting mistakes, seeking feedback, and empowering their employees to make decisions and take risks, are competencies that leaders exhibit when committed to the goals of the organizations (15). Senge’s theory has been applicable to different organizational settings (15, 16).

Besides *learning organizations theory*, emphasis was also made on elements of *transformational leadership*, such as *shared visioning*, *motivating (self and others)*, *creativity to seek new ways*, *seeking opportunities in the face of risk*, *networking*, and *effective communication* (17). A significant part of leadership development has also allocated to theories and practices related to *Leadership from inside out* model. Almost all training occasions have been based on *experiential learning* (18) journey to maximize experiencing and uptakes among the adult and professional audience of nutrition leadership initiative.

### Program vision, mission, and values

The nutrition leadership initiative is intended to build national and regional platforms and opportunities for nutrition professionals to be empowered and co-create transformational leadership solutions in a constantly changing world. As Senge once said, “In the long run, the only sustainable source of competitive advantage is your organization’s ability to learn faster than its competition.”

Given the above, the nutrition discipline shall be a learning organization, if it is expected to overcome complex challenges that act as barriers to human well-being and national development. Accordingly, nutrition professionals are supposed to be committed, i.e., motivated and confident (19), and well-equipped with knowledge and skills required to fight against malnutrition, food safety challenges, and diet-related non-communicable diseases. Therefore, leadership development programs shall be able to inspire, empower, and connect nutrition professionals with the ultimate goal of making a better community and future. It has been considered as a journey in which an individual, a team, or an organization takes the responsibility to improve self and others, say, for health and progress. Respecting diversity, empowerment, motivation, inspiration, sustainability, and trust are foundations of such an endeavor.

### Sensitization and advocacy

There have been two main grounds to advocate leadership development programs among nutrition professionals in Iran. We have taken the opportunities of 4 biannual national nutrition congresses since 2008 to sensitize attendees (more than 2000 on average; the last nutrition congress recorded registration of 3300 attendees on the first day), and to introduce the leadership concept through plenary presentations. Organizing training events in partnership with different stakeholders have also acted as a powerful advocacy endeavor. We may add research projects as the third potential occasions in which forums are built for raising the issue and exchanging ideas.

### Training activities

Since 2009, a number of leadership development activities for nutrition professionals, as well as present and promising leaders, were made, which briefly introduced above. Table 1 wraps up the training events that have been developed and administered in the last 6 years.

**Table 1:** **Nutrition leadership development initiatives in Iran and the Middle-East region**.

	Year	Program name	Target/description
1	2009	Iranian Food and Nutrition Leadership Program (IFNLP)	Young food and nutrition professionals from different sectors as present or promising future leaders in the field. The first and the second workshops were held in 2009 and 2014, respectively
2	2010	One vision one voice	Present nutrition leaders in academe and Ministry of Health. Two workshops were held in 2010
3	2011	Leadership training of Ph.D. students in Nutrition Programs (as part of the 4-U core course on Managing Nutrition programs)	Ph.D. students in Shiraz University of Medical Sciences started the initiative. Other leading nutrition departments, i.e., Shahid Beheshti, Iran and Tehran Universities of Medical Sciences have joined the initiative. At present, the training program is separately offered at each University in each educational year
4	2011	ATA’s Nutrition Leadership Program	Young nutrition members to the professional NGO Iranian Nutrition Society (ATA) participated in 12 self-funded workshops at 4 different levels from 2011 to 2013
5	2012	The Middle-East Nutrition Leadership Program (MENLP)	The first workshop was held with nutrition and health officers of EMRO-WHO member countries. The second workshop in 2015 was organized for regional UNICEF nutrition and health staff. Local nutrition professionals form different sectors were also included in both trainings
6	2013	Leadership Development among Hospital Dieticians	Clinical nutritionists from selected hospitals in the Capital city and selected provinces participated in the Nestle-funded training
7	2014	Leadership Development among Community Nutrition and Health Officers	Community health and nutrition officers in a selected province participated in a leadership training. The initiative has been decided to cover provincial community nutrition managers

Besides programs listed in Table 1, leadership capacity-building workshops have successfully been organized for some other groups of professionals, such as physicians, hospital staff, health officers, and food and pharmaceutical company employees and managers.

## Lessons Learned

Leadership development of nutrition professionals in the last 6 years has provided a number of invaluable lessons, including but not limited to the following.

1. ***Leadership development has been welcomed by nutrition community***
2. Leadership training programs are adaptable to different clients as well as different career stages, mainly because participants easily connect to, reflect on, and integrate the issue based on their goals and background. It is especially evident when participants practice self-leadership skills. Moreover, participants tend to name it as “a totally different and amazing type of experience within already routine activities in life and at workplace.”
3. ***Development of leadership competencies at younger ages seems more effective***
4. While there is no age boundary to education and learning, but leadership skills, as many other competencies, is better to be developed at earlier life and career stages. Needless to mention that, since development of social intelligence as a key determinant of leadership is an age-dependent process, leadership training in older ages shall also be encouraged.
5. ***Learning by experiencing significantly lasts longer***
6. Stories, games, and different individual and interactive team assignments have been very effective for our adult and professional participants in leadership workshops. Seminars can provide introductions, theoretical background, and working definitions, but can never replace the deeper understanding and learning levels participants experience by “doing” themselves.
7. ***Participant–Facilitator” interaction is a shared developmental journey***
8. An interactive leadership workshop is far beyond one-way teaching experience happening in a technical class session. Indeed, facilitators are also being developed in a leadership journey through exchange of thoughts and feelings, and even observing participants’ interactions. Such a co-creation and co-development is unique in every single “participant–facilitator” interaction.
9. ***Alumni are the moving engines of leadership programs***
10. Sustainability of leadership programs and keeping the momenta of personal and organizational transformation require continuous alumni engagement, as the true change makers. Therefore, alumni networking and activity shall find its place at the core of any leadership development program.
11. ***A common purpose can best lead nutrition community***
12. Nutrition scientists and practitioners, as other professionals, need a shared vision as the “common purpose”. That common purpose, when coupled with an effective networking exercise among the empowered human resource, can inspire and lead the whole system towards the desired direction. Food and security, for instance, has the power to act as a strong common purpose among professionals working in the field of nutrition. It also provides a common ground and language for a diverse range of stakeholders, disciplines, and sectors which are active in a wide spectrum, from human right and well-being to national development.

## Road to Future

We see ourselves at the beginning of a capacity-building journey, and a jump-start point for leadership empowerment of nutrition professionals in this region. Research resides at the core of this journey as a key to unlock many uncertainties and challenges that we have in this new field. A strong monitoring and evaluation component shall be developed and integrated into the program, so that we can systematically learn deeper and beyond what we obtain through satisfaction level feedbacks (20). Moreover, leadership development is an increasing demand, which necessitates more facilitators and coaches to step into the area. Therefore, training of the trainers (ToTs) shall be rigorously planned. We started to establish effective platforms for both regional (MENLP) and local initiatives to better interact with our target audience and alumni networks. While there has been some initial exercises in this regard (11), more organized efforts and sustainable innovative suggestions are required.

## Conclusion

Investing on professional human resource empowerment in different branches of nutrition discipline together with networking will translate into better intra- and inter-disciplinary understanding, collaboration, and concerted action. Interfacing with a diverse range of disciplines and sectors in nutrition science and practice turns this investment into success. Leadership development has the potential to facilitate creating shared vision, trust building, enhancing personal motivation, and maximizing group relations, as well as organizational connectedness and performance. Leadership trainings will equip professionals with required competencies, and unify them to lead the changes that they are supposed to make in their constantly changing environment.

## Conflict of Interest Statement

The authors declare that the research was conducted in the absence of any commercial or financial relationships that could be construed as a potential conflict of interest.

## References

[1] Yukl GA Leadership in Organization. 7th ed Upper Saddle River, NJ: Pearson Prentice-Hall (2010).

[2] Robinson-Hickman G Sorenson GJ The Power of Invisible Leadership. 1st ed Thousand Oaks, CA: SAGE (2014).

[3] Hughes R Time for leadership development in the public health nutrition workers. Public Health Nutr (2009) 12(8):1029 10.1017/S1368980009990395 19570300

[4] Margetts B Leadership and responsibility in public health nutrition: time to get serious. Public Health Nutr (2006) 9(5):533–9. 10.1079/PHN2006978

[5] McCollum G Leadership in nutrition and dietetics: today’s wisdom for tomorrow’s leader. J Acad Nutr Diet (2014) 114(Suppl 1):S3 10.1016/j.jand.2014.03.007 24725523

[6] Kvaskoff M McKay SD Education: scientists need leadership training. Correspondence. Nature (2014) 506:159 10.1038/506159c 24522590

[7] Seeliger JC Scientists must be taught to manage. Nature (2012) 483:511 10.1038/483511a 22460862

[8] Davari A Rashidi A Development of leadership competencies among food and nutrition scientists: a road to greatness. Nutr Food Sci Res (2015) 2(2):1–2.

[9] Ghassemi H A Note on Development of New Graduate Programs in the Nutrition Sciences in Iran. Tehran: The joint project of Ministry of Health and Medical Education and The World Bank (2004).

[10] Davari A Needs assessment and preliminary program development to improve leadership skills among food technologists in Iran. Thesis Submitted for Master Degree in the Food Science and Technology. Tehran: School of Nutrition Sciences and Food Technology, Shahid Beheshti University of Medical Sciences (International Branch) (2009) [in Farsi].

[11] Davari A Drew T Pick P Reynolds S Vano N Middle East Nutrition Leadership Program – Residential Leadership Conference: Evidenced-based Program Design. as part of the course LEAD500, School of Leadership and Education Sciences, California: University of San Diego (2014).

[12] Davari A Rashidi A Zali MR Ehsani MR Leadership capabilities among Iranian food science and technology specialists: educational needs assessment. Iran J Nutr Sci Food Technol (2013) 8(3):269–78 [in Farsi].

[13] Senge PM The Fifth Discipline: The Art and Practice of the Learning Organization. New York, NY: Doubleday/Currency (2006).

[14] Mathison S Why triangulate? Educ Res (1988) 17(2):13–7. 10.3102/0013189X017002013

[15] Goh S Richards G Benchmarking the learning capability of organizations. Eur Manag J (1997) 15(5):575–83. 10.1016/S0263-2373(97)00036-4

[16] du Plessis D du Plessis M Millet B Developing a learning organization: a case study. J Manag Pract (1999) 2(4):71–94.

[17] Lowe KB Kroeck KG Sivasubramaniam N Effectiveness correlates of transformational and transactional leadership: a meta-analysis review of the MLQ literature. Leadersh Q (1996) 7(3):385–415. 10.1016/S1048-9843(96)90027-2

[18] Kolb DA Experiential Learning: Experience as the Source of Learning and Development. Englewood Cliffs, NJ: Prentice-Hall, Inc (1984).

[19] Blanchard KH A situational Approach to Managing People. Blanchard Training and Development, Inc (1985).

[20] Kirkpatrick DL Kirtpatrick JD Evaluating Training Programs: The Four Levels. 3rd ed San Francisco, CA: Berrett-Koehler Publishers, Inc (2006).

